# The effect of baseline cognition and delirium on long-term cognitive impairment and mortality: a prospective population-based study

**DOI:** 10.1016/S2666-7568(22)00013-7

**Published:** 2022-04

**Authors:** Alex Tsui, Samuel D Searle, Helen Bowden, Katrin Hoffmann, Joanne Hornby, Arley Goslett, Maryse Weston-Clarke, Lee Hamill Howes, Rebecca Street, Rachel Perera, Kayvon Taee, Christoph Kustermann, Petronella Chitalu, Benjamin Razavi, Francesco Magni, Devajit Das, Sung Kim, Nish Chaturvedi, Elizabeth L Sampson, Kenneth Rockwood, Colm Cunningham, E Wesley Ely, Sarah J Richardson, Carol Brayne, Graciela Muniz Terrera, Zoё Tieges, Alasdair MacLullich, Daniel Davis

**Affiliations:** Medical Research Council Unit for Lifelong Health and Ageing at University College London, University College London, London, UK; Medical Research Council Unit for Lifelong Health and Ageing at University College London, University College London, London, UK; Geriatric Medicine, Dalhousie University, Halifax, NS, Canada; Medical Research Council Unit for Lifelong Health and Ageing at University College London, University College London, London, UK; Medical Research Council Unit for Lifelong Health and Ageing at University College London, University College London, London, UK; Medical Research Council Unit for Lifelong Health and Ageing at University College London, University College London, London, UK; Medical Research Council Unit for Lifelong Health and Ageing at University College London, University College London, London, UK; Medical Research Council Unit for Lifelong Health and Ageing at University College London, University College London, London, UK; Medical Research Council Unit for Lifelong Health and Ageing at University College London, University College London, London, UK; Medical Research Council Unit for Lifelong Health and Ageing at University College London, University College London, London, UK; Medical Research Council Unit for Lifelong Health and Ageing at University College London, University College London, London, UK; Medical Research Council Unit for Lifelong Health and Ageing at University College London, University College London, London, UK; Medical Research Council Unit for Lifelong Health and Ageing at University College London, University College London, London, UK; Medical Research Council Unit for Lifelong Health and Ageing at University College London, University College London, London, UK; Medical Research Council Unit for Lifelong Health and Ageing at University College London, University College London, London, UK; Medical Research Council Unit for Lifelong Health and Ageing at University College London, University College London, London, UK; Medical Research Council Unit for Lifelong Health and Ageing at University College London, University College London, London, UK; Medical Research Council Unit for Lifelong Health and Ageing at University College London, University College London, London, UK; Medical Research Council Unit for Lifelong Health and Ageing at University College London, and Division of Psychiatry University College London, London, UK; Medical Research Council Unit for Lifelong Health and Ageing at University College London, University College London, London, UK; Geriatric Medicine, Dalhousie University, Halifax, NS, Canada; School of Biochemistry & Immunology, Trinity Biomedical Sciences Institute, Dublin, Ireland; Critical Illness, Brain Dysfunction, and Survivorship Center, Tennessee Valley Veterans Affairs Geriatric Research Education Clinical Center and Vanderbilt University Medical Center, Nashville, TN, USA; AGE Research Group, Translational and Clinical Research Institute, Newcastle University, Newcastle, UK; Department of Public Health and Primary Care, University of Cambridge, Cambridge, UK; Edinburgh Dementia Prevention University of Edinburgh, Edinburgh, UK; Edinburgh Dementia Prevention and Geriatric Medicine, Edinburgh Delirium Research Group, Usher Institute University of Edinburgh, Edinburgh, UK; SMART Technology Centre, Glasgow Caledonian University, Glasgow, UK; Edinburgh Dementia Prevention and Geriatric Medicine, Edinburgh Delirium Research Group, Usher Institute University of Edinburgh, Edinburgh, UK; Medical Research Council Unit for Lifelong Health and Ageing at University College London, University College London, London, UK

## Abstract

**Background:**

There is an unmet public health need to understand better the relationship between baseline cognitive function, the occurrence and severity of delirium, and subsequent cognitive decline. Our aim was to quantify the relationship between baseline cognition and delirium and follow-up cognitive impairment.

**Methods:**

We did a prospective longitudinal study in a stable representative community sample of adults aged 70 years or older who were registered with a Camden-based general practitioner in the London Borough of Camden (London, UK). Participants were recruited by invitation letters from general practice lists or by direct recruitment of patients from memory clinics or patients recently discharged from secondary care. We quantified baseline cognitive function with the modified Telephone Interview for Cognitive Status. In patients who were admitted to hospital, we undertook daily assessments of delirium using the Memorial Delirium Assessment Scale (MDAS). We estimated the association of pre-admission baseline cognitive function with delirium prevalence, severity, and duration. We assessed subsequent cognitive function 2 years after baseline recruitment using the Telephone Interview for Cognitive Status. Regression models were adjusted by age, sex, education, illness severity, and frailty.

**Findings:**

We recruited 1510 participants (median age 77 [IQR 73–82], 57% women) between March, 2017, and October, 2018. 209 participants were admitted to hospital across 371 episodes (1999 person-days of assessment). Better baseline cognition was associated with a lower risk of delirium (odds ratio 0·63, 95% CI 0·45 to 0·89) and with less severe delirium (–1·6 MDAS point, 95% CI –2·6 to –0·7). Individuals with high baseline cognition (baseline Z score +2·0 SD) had demonstrable decline even without delirium (follow-up Z score +1·2 SD). However, those with a high delirium burden had an even larger absolute decline of 2·2 SD in Z score (follow-up Z score –0·2). Once individuals had more than 2 days of moderate delirium, the rates of death over 2 years were similar regardless of baseline cognition; a better baseline cognition no longer conferred any mortality benefit.

**Interpretation:**

A higher baseline cognitive function is associated with a good prognosis with regard to likelihood and severity of delirium. However, those with a high baseline cognition and with delirium had the highest degree of cognitive decline, a change similar to the decline observed in individuals with a high amyloid burden in other cohorts. Older people with a healthy baseline cognitive function who develop delirium stand to lose the most after delirium. This group could benefit from targeted cognitive rehabilitation interventions after delirium.

## Introduction

The clinical importance of delirium, which presents with acute changes in arousal, inattention, and global cognitive impairment affecting one in four older (≥70 years) inpatients, is well established.^1^ Delirium is distressing to patients and to those who care for them, and it is harmful.^2,3^ This harm extends beyond short-term events such as injurious falls and includes a greater risk of death and long-term cognitive impairment.^4,5^ Yet, to advance understanding of how to improve care, an expert review of international delirium research emphasised that “particular vulnerabilities predicting the negative long-term outcomes of delirium will be key to identifying at-risk patient groups and to developing targeted therapies”.^6^

Cognitive decline after delirium has been shown in several settings, including in critical care, perioperatively, and in population cohorts aged 50 years or older.^7–12^ Understanding how this arises has proved challenging because of two methodological issues. First, studies have rarely established baseline cognition, so any cognitive impairment observed at follow-up might be confounded by undiagnosed pre-existing cognitive impairment.^7,13^ Second, delirium has been retrospectively ascertained, so detailed information on the features of delirium is absent and it is substantially underdiagnosed.^10–12^ Although there are established data on elective surgical cohorts, on the whole, patients in such cohorts are less frail and are less likely to be living with dementia than patients who are admitted to hospital for unselected unscheduled surgery or acute care.^14^ Contemporary population-based studies of delirium and cognition are needed to determine the key vulnerabilities predicting negative long-term cognitive outcomes from delirium across the whole spectrum of older people presenting to acute care.

To understand the relationship between baseline cognition, delirium, and outcomes of delirium, we characterised baseline cognitive function in a stable community sample of older people. Then, at each acute admission to hospital we systematically: (1) assessed the point prevalence, severity, and duration of delirium; (2) assessed the associations of these estimates of delirium with baseline cognition; and (3) assessed the association between delirium and long-term cognitive and mortality outcomes at 2 years after baseline assessment and how these associations were modified by baseline cognition. We hypothesised first that baseline cognition would affect delirium risk, severity, and duration; and second, that baseline cognition would interact with cumulative delirium exposure, resulting in different long-term cognitive and mortality outcomes.

## Methods

### Study design and participants

**Figure 1:**
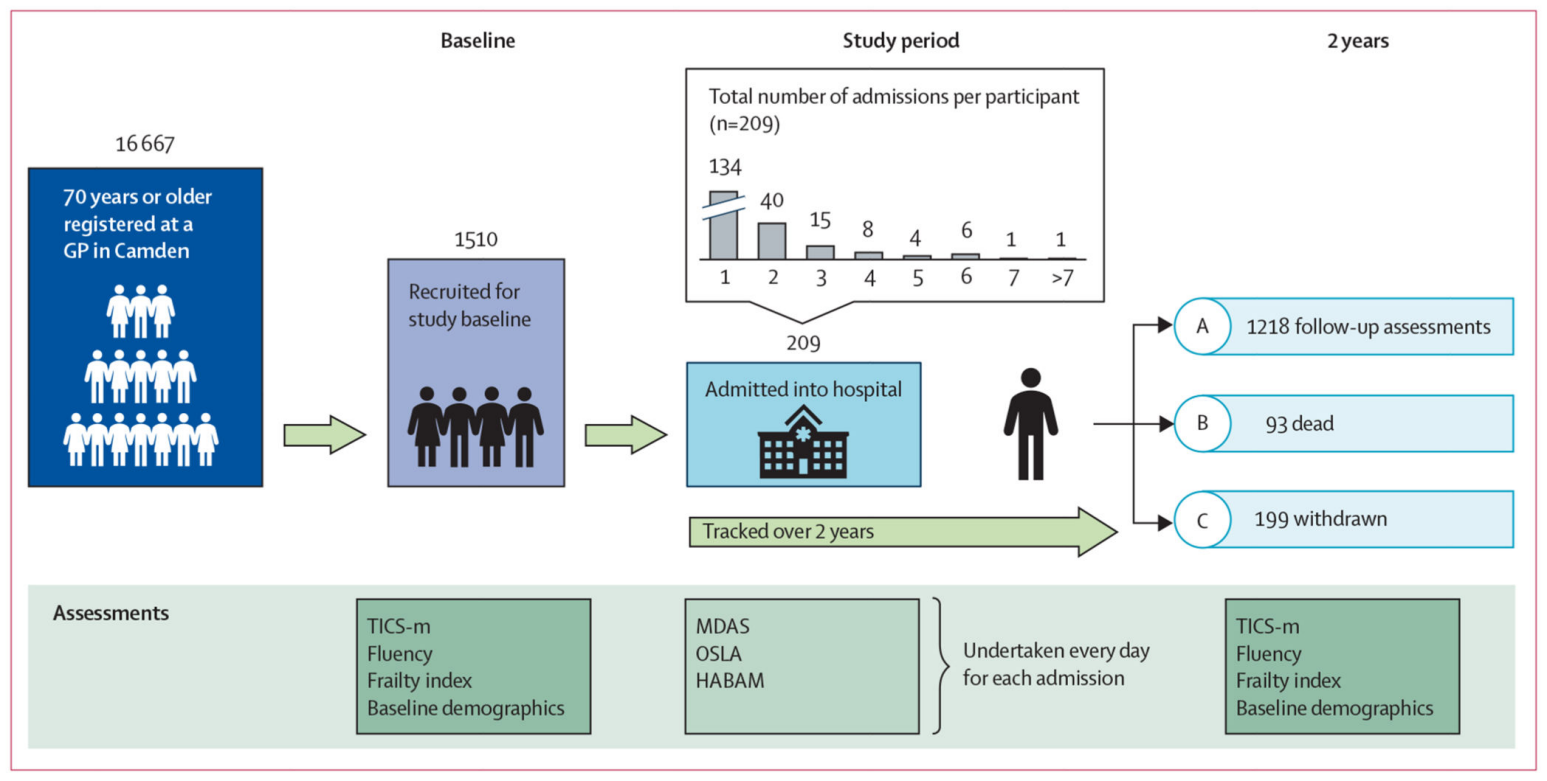
Infographic showing patient recruitment and study timeline GP=general practitioner. HABAM=Hierarchical Assessment of Balance and Mobility. MDAS=Memorial Delirium Assessment Scale. OSLA=Observational Scale of Level of Arousal. TICS-m=modified Telephone Interview for Cognitive Status.

The Delirium and Population Health Informatics Cohort (DELPHIC) is a prospective longitudinal study of adults aged 70 years or older in the London Borough of Camden (London, UK), a region with 260 000 residents (figure 1).^15^ The UK National Health Service (NHS) provides more than 95% of health care, and Camden is served by a single primary care system and two acute hospitals. This report is a planned analysis of approximately the first 1500 participants.

Eligible participants were aged 70 years or older and were registered with a Camden-based general practitioner. Using codes in primary care records, we excluded those with severe hearing impairment, aphasia, an inability to speak English sufficiently to undertake any basic cognitive assessment, or those in the terminal phase of an illness. We invited individuals to enrol by letter from general practice lists, augmented by the direct recruitment of patients from memory clinics and those recently discharged from secondary care in an 8:1:1 ratio. We adopted this strategy to increase the chance of including a wider range of health states in the recruited sample. Individuals, or their nominated proxies, gave consent or agreement to take part in accordance with the Mental Capacity Act 2005, and researchers were trained through the National Institute of Health Research’s Good Clinical Practice certification.

All team members received standard training from senior researchers (led by HB). Community assessments (at baseline and follow-up) were performed by graduate researchers, in pairs to ensure a degree of cross-validation. The hospital assessments were performed by registered health-care professionals (HB, occupational therapy; KH, registered nurse). All clinical cases were discussed on a once per week basis with other senior clinicians on the team. The protocol received approval from an NHS Research Ethics Committee (16/LO/1217) and the Health Research Authority (IRAS 164446). All participants or their proxies gave informed consent before taking part.

### Procedures

Baseline cognitive assessments were performed in all participants. Follow-up delirium assessments were done only in those participants who were admitted to hospital. Assessment interviews, including cognitive function assessments, were performed at baseline and repeated 2 years after initial recruitment, although we delayed the follow-up appointment if an individual had recently been admitted to hospital to reduce the chance that assessments were confounded by persistent delirium. Most assessments took place by telephone; for participants with substantial hearing impairment not captured in the primary care record who still wished to participate, we arranged home visits. Cognitive function was the primary measure, assessed using the modified Telephone Interview for Cognitive Status (TICS-m), which covers orientation, repetition, naming, and immediate and delayed recall of a 10-item non-semantically related word list.^16^ We supplemented this assessment with two tasks of verbal fluency adapted from the Addenbrooke’s Cognitive Examination, in which participants were asked to generate words beginning with the same letter (P) and to generate as many different animals as they could.^17^ At the baseline assessment and at the one follow-up interview, we recorded: sociodemographic factors, index of multiple deprivation, general health, comorbidities, medications, health behaviours, hearing, vision, quality of life, dental health, continence, falls, depression, and personal and instrumental activities of daily living. Frailty was quantified as a cumulative index of health deficits (0–1), derived using 28 items drawn from the baseline assessment and calculated in line with standard procedures and with the same coverage of health and functional domains.^18^ However, we excluded cognitive items from the frailty index to avoid collinearity with the primary cognitive measure. We were notified about participant deaths from the NHS Spine, a statutory register for all deaths in England, and we cross-referenced these with local hospital electronic records systems. AT, SDS, HB, KH, JH, AG, MW-C, LHH, RS, RP, KT, CK, PC, BR, FM, DDas, and SK collected these data.

All participants admitted to either of the two hospitals in Camden were automatically flagged through daily electronic alerts and reviewed in person each day (from Monday to Friday) from the day of admission by a researcher. At each hospital assessment, we evaluated participants for changes in amount of arousal or cognitive or physical function using the Memorial Delirium Assessment Scale (MDAS), Observational Scale of Level of Arousal (OSLA), and the Hierarchical Assessment of Balance and Mobility (although this last assessment does not form part of this analysis).^19–21^ Additional information on acute causes, medications, and laboratory findings were recorded. Our primary measure of illness severity was the National Early Warning Score (NEWS).^22^ NEWS aggregates physiological disturbances (heart rate, blood pressure, respiratory rate, oxygen saturations, supplemental oxygen, and alertness). The original NEWS scale used here ranges from 0 to 20, where the risk of immediate deterioration increases with higher scores. Any score higher than 4 should trigger a clinical review for an escalation of care.

Ascertainment of delirium used the Diagnostic and Statistical Manual of Mental Disorders (DSM)-IV criteria as the primary outcome, because it is the most widely used definition and allows for comparative estimates with other studies.^1^ Delirium was ascertained by a consensus panel (AT, SDS, HB, KH, and DDav) for every day of hospital admission using all available information. On each day, delirium was established to be present if individuals met criteria A (disturbance of consciousness), B (change in cognition or perception, or both), and C (acute and fluctuates). By virtue of their inpatient admission, all participants were deemed to fulfil criterion D (physiological consequence of a general medical condition).

### Statistical analysis

Delirium point prevalence was defined as the proportion of participants who met criteria for all DSM-IV components within each 24-h period. Delirium severity was defined as MDAS assessments of the 10 domains of delirium symptoms (each scored out of 3) to give a 30-point measure of delirium severity.^19^ Delirium duration was calculated as the total number of delirium days experienced over the study period (across multiple admissions where relevant). Delirium burden was quantified by combining a measure of duration and severity through summing up daily MDAS scores (expressed as MDAS points multiplied by number of days assessed). All delirium assessments were done only in participants admitted to hospital. We defined individuals with scores greater than or equal to the sample median as having a high delirium burden, and those with scores below the median as having a low delirium burden. All other participants (who received a score of 0) were classified as no delirium burden. This approach allows for a simpler interpretation and is more likely to be robust, although this measure will not necessarily translate to other cohorts.

For cognitive function, both baseline (exposure) and follow-up (outcome) used the composite cognitive score (TICS-m scored out of 53 points and verbal fluency scored out of 14 points, standardised as [composite score–mean]/SD). The standardised Z score is normally distributed with a mean of 0; +2·0 SD and –2·0 SD include approximately 95% of values. Mortality was defined as any deaths occurring before follow-up, in hospital or in the community.

Missing data were handled in the following manner. In-hospital assessments missing because they were done on a weekend or public holiday (missing at random) were forward filled (data from Friday carried to Saturday) and backward filled (Sunday carried data from Monday, and public holidays were usually backward filled but sometimes forward filled) in 24-h intervals for up to 4 days. For backward filling, this approach has the advantage of automatically carrying over information from the next working day’s chart review. Otherwise, data were assumed to be missing at random. To understand factors associated with attrition, we performed logistic regression where the outcome was loss to follow-up, adjusted by baseline cognition, age, sex, frailty, and mean NEWS.

For baseline cognition and subsequent delirium, models were estimated using mixed-effects regression (logistic regression for delirium point prevalence and linear regression for delirium severity). We used negative binomial regression to estimate the differences in delirium duration, calculating days in delirium (count data) over the length of the hospital stay. All analyses were adjusted by age, sex, baseline cognition, education, frailty index, and NEWS.

For delirium and long-term cognitive impairment or mortality, linear regression estimated a cognitive change at 2 years after baseline assessment. We performed Cox regression for proportional hazards of death, including incident delirium (yes or no) or delirium burden (none, low, or high) as the independent variable. We included a multiplicative term to quantify any interactions between baseline cognition and degree of delirium. Again, we included age, sex, baseline cognition, education, frailty index, and mean NEWS as factors.

Our a priori power calculations suggested that a minimum of 11% of the cohort would need to be admitted to hospital, and 70% be followed up, to detect a clinically significant change in long-term cognition (six modified TICS-m points, or approximately 0·3 SD in Z score).^15^ We used Stata 17.0 for all analyses.

### Role of the funding source

The funder of the study had no role in study design, data collection, data analysis, data interpretation, or writing of the report.

## Results

Of the 1510 participants recruited between March 1, 2017, and Oct 31, 2018, the median age was 77 (IQR 73–82), and 865 (57%) were women (table 1). We undertook home assessments in 32 participants who could not use a telephone. We sent 24 162 single invitations in batches, from which we recruited 1311 participants (5% response), with an additional 39 participants (3% of the final sample) recruited from the memory clinic and 141 participants (9% of the final sample) recruited from secondary care, all of whom were deemed eligible to participate and were included in analyses. Compared with the demographics of Camden, the sample was well matched by age and index of multiple deprivation scores, and absolute differences were small except for ethnic representation, because there was a higher proportion of White participants than for Camden as a whole (appendix pp 1, 3). Out of a score of 67, baseline cognition had a mean of 50 (SD 8·0) and 90% of values were between 35 and 60 points indicating no floor or ceiling effects. In terms of formal clinical diagnoses, 51 (3%) participants had been diagnosed with dementia and a further 13 participants had mild cognitive impairment.

Over the study period (median follow-up, 2·5 years [IQR 2·6–2·9], total of 3842 person-years until July 31, 2021), 209 participants (14%) were admitted to hospital in 371 episodes, representing 1999 person-days of assessment, 40 (2%) of which were in critical care. Individuals admitted to hospital only once accounted for 114 (55%) hospital participants. The remaining 95 participants were admitted multiple times (median number of recurrent admissions was 2 [IQR 2–4]). At 2 years, 1218 (81%) of 1510 participants had repeat cognitive assessments, 93 (6%) participants had died, and 199 (13%) were lost (or withdrew) to follow-up.

Over the course of the study, 115 (8%) of individuals experienced delirium at least once. On any given day (point prevalence), an average of 29% of participants admitted to hospital fulfilled the DSM-IV criteria for delirium. At any assessment, participants met DSM-IV criterion A in 1379 (69%) of 1999 assessments, criterion B in 1359 (68%) assessments, and criterion C in 820 (41%) assessments. Measures contributing to criterion A included abnormal OSLA scores (620 [31%] of 1999 assessments) and an inability to perform the Months of the Year Backward test (259 [13%]). Features fulfilling criterion B included short-term memory impairment in 620 (31%) assessments and perceptual disturbance in 256 (13%) assessments. There was evidence of fluctuation (criterion C) in OSLA or MDAS scores (differing from the previous assessment by ≥1 SD) in 100 (5%) of assessments. New severe sleep–wake cycle disturbance was present in 397 (20%) assessments.

In those admitted to hospital, the median MDAS score was 7 (IQR 3–12) of 30 points. The delirium burden (cumulative MDAS scores) had a median of 26 (IQR 20–197) MDAS points multiplied by number of delirium days, equivalent to approximately 2 days of mild delirium in the context of the original MDAS validation study (mild delirium burden <16, moderate delirium burden 16–22, and severe delirium burden >22).^19^

**Figure 2:**
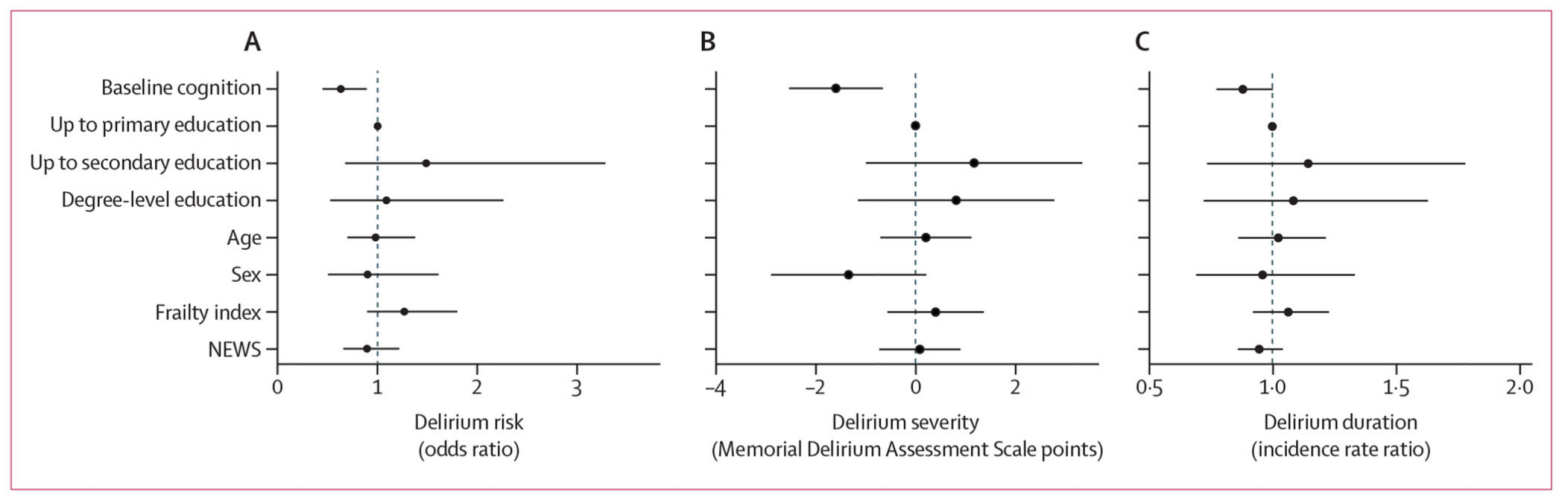
Delirium risk, severity, and duration when adjusted NEWS=National Early Warning Score.

After adjustment by age, sex, education, NEWS, and frailty, the risk of delirium was lower in participants with better baseline cognition (odds ratio [OR] 0·63 per 1 SD higher baseline cognition, 95% CI 0·45–0·89, p=0·009; figure 2A; appendix p 2). Educational attainment was not associated with any delirium outcome. Better baseline cognition was associated with less severe delirium (–1·6 MDAS points per 1 SD higher baseline cognitive score,–2·6 to –0·7, p=0·001; figure 2B; appendix p 2). Better baseline cognition was also associated with a shorter delirium duration (incidence rate ratio 0·88 per 1 SD increase in baseline cognition 0·77–1·00, p=0·054; figure 2C; appendix p 2). Clinically, this translates to a patient with one SD better baseline cognition having one fewer day of delirium per week of illness than patients of similar frailty and illness severity.

We followed up 1218 (81%) of the cohort, with 34 participants being seen as home visits. The shortest duration between the last admission to hospital and follow-up was 25 days (the next four shortest intervals were 36, 37, 57, and 82 days). Individuals with lower baseline cognition were more likely to be lost to follow-up (OR –0·82 per 1 SD increase in baseline cognitive score; 95% CI –1·28 to –0·38; p<0·0001). Of those lost to follow-up, 28 (14%) participants had been admitted to hospital, 18 (9%) of whom had delirium.

Delirium at any point was associated with a worse long-term cognitive decline (0·35 SD deficit in follow-up cognitive score, 95% CI 0·63–0·07, p=0·016). We did not undertake clinical examinations to define incident dementia, but 179 individuals had Z scores less than –2·0 from the baseline assessment.

**Figure 3:**
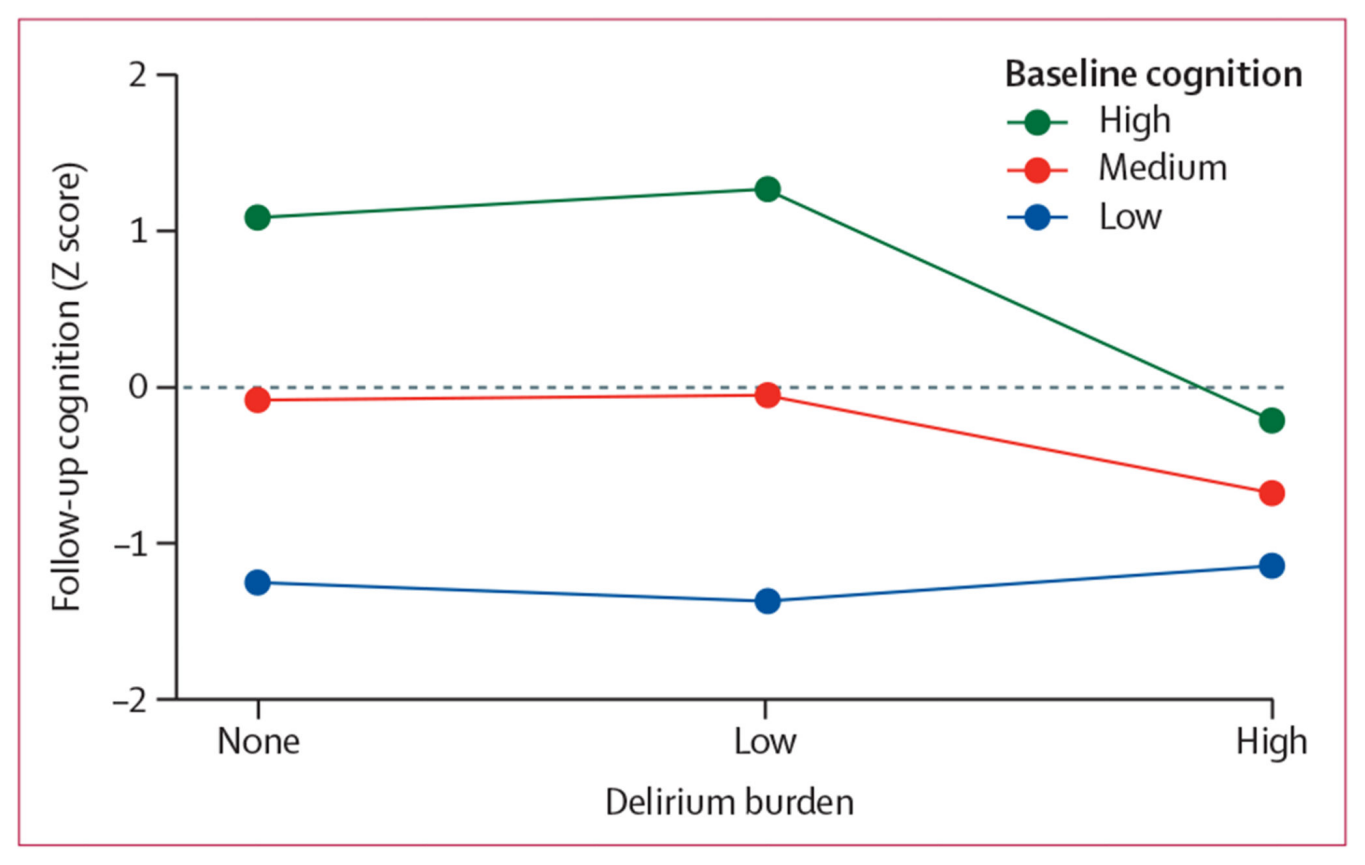
Association between delirium burden and follow-up cognition by baseline cognition

In the whole cohort, high, but not low, delirium burden was associated with worse follow-up cognition: those with high delirium burden (ie, with 26 or more cumulative MDAS points) had a 0·60 SD deficit in follow-up cognitive scores (95% CI –0·93 to –0·26, p<0·0001), compared with participants of similar baseline cognition, frailty, and illness severity who did not have any delirium (figure 3; appendix p 2). Educational attainment was associated with better follow-up cognition (appendix p 2).

Beyond the general association between delirium burden and follow-up cognition, there were different effect sizes according to baseline cognition (interaction term p=0·016; figure 3; table 2). Those with low baseline cognition had similar scores at follow-up regardless of delirium exposure (Z score –1·3 with no delirium burden, –1·4 with low delirium burden, and –1·2 with high delirium burden). Individuals with high baseline cognition—namely, those starting at +2·0 SD in Z score—had a demonstrable decline in cognition even without delirium (follow-up Z score, +1·17). However, patients with high baseline cognition who had a high delirium burden had an even larger absolute decline of 2·2 SD in Z score (follow-up Z score, –0·2; figure 3).

**Figure 4:**
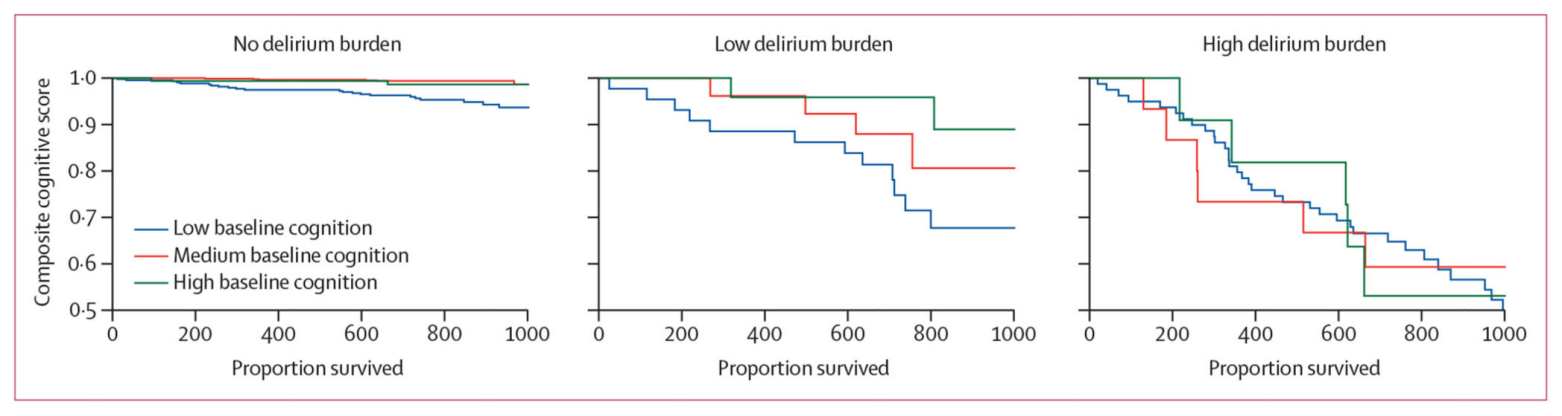
Association between baseline cognition and survival, by delirium burden

In the 93 (6%) participants who died, the median follow-up was 444 days (IQR 282–747). 63 participants died in hospital, and 30 participants died in community settings. Of those who died in hospital, 18 participants had a low delirium burden and 45 participants had a high delirium burden during the follow-up interval. Overall, delirium was associated with increased mortality (hazard ratio 6·4, 95% CI 3·2–12·7, p<0·0001). In the absence of delirium, mortality was lower in those with high baseline cognition (0·64, 0·49–0·83, p<0·0001; appendix p 2). However, individuals with better baseline cognition and a high delirium burden had a higher risk of death compared with those with a low baseline cognition and high delirium burden (1·6, 1·1–2·2, p_interaction_=0·016; appendix p 2). People with a high delirium burden and high baseline cognition had a similar mortality risk compared with those with high delirium burden and low baseline cognition (ie, baseline cognition makes no difference; figure 4).

## Discussion

Our study showed that a higher baseline cognition is linked with lower risk of delirium, and in those who developed delirium, associated with a shorter and less severe delirium, even after accounting for acute illness severity and frailty. A high delirium burden, defined by duration and severity, negatively affected both follow-up cognition and mortality. Notably, those with higher baseline cognition had the most significant change, with at least 1·0 SD cognitive decline attributable to delirium. Taken together, these findings suggest those with a healthy baseline cognitive function who develop delirium are at much higher risk of new cognitive impairment, standing to lose the most after delirium. This group might benefit the most from intensive delirium management and post-delirium follow-up, but the mechanisms underlying this association are unclear.

In high functioning individuals, a 1 SD change in cognition over 2 years is notable. It is similar to rates of decline observed in other high-risk groups in longitudinal studies of brain ageing. Examples of amyloid-related decline in healthy individuals range from 0·15 to 0·35 SD per year.^23^ In the Alzheimer’s Dementia Neuroimaging Initiative, those positive for amyloid by PET imaging or in cerebrospinal fluid samples also had a 1 SD cognitive decline, emerging over 3 years.^24^ Our primary cognitive measure (baseline cognition) was normally distributed, without floor or ceiling effects. Therefore, although regression to the mean was evident in the follow-up scores of participants not experiencing delirium, the asymmetry of a greater decline in cognition affecting those with a higher baseline cognition is an additional effect to any regression to the mean. With regard to understanding why individuals with a better baseline cognition were susceptible to cognitive decline and death associated with delirium, it is possible that the physiological precipitants of delirium in these individuals somehow had a more direct neurological effect than in participants with worse baseline cognition. NEWS values were not higher in those with better cognition who subsequently died; although our analyses adjusted for acute illness severity, it might be that NEWS is an inadequate measure in older people. Nonetheless, this finding echoes a previous result showing a disproportionate effect of delirium on healthier individuals or on those without a history of dementia.^25^ If delirium represents a better indication of the severity of acute illness in this subgroup, de novo presentations should be regarded as especially concerning.

Our related study, Delirium and Cognitive Impact in Dementia, showed that delirium was associated with subsequent dementia in a dose-dependent manner.^26^ Collectively, the broader findings align with experimental data from animal models showing that increasing grades of previous neurodegeneration lead to more severe and long-term delirium signs when challenged with a standard inflammatory stimulus.^27^ Evidence from neuropathological population studies shows that many individuals develop delirium without a specific pathology.^28^ Similarly, frailty appears to modify the clinical expression of dementia neuropathology.^29^ We now show that baseline cognition positively influences delirium burden; yet, when delirium becomes established in those with high baseline cognition, outcomes are substantially worse. In this way, it is clear the general relationship between cognitive impairment, delirium, and frailty reaches beyond the classic framework of dementia as principally a chronic neurodegenerative condition. Indeed, dissecting delirium components beyond a unitary construct (examining cause and specific phenomenological links to underlying brain pathology) has the potential to enhance understanding of the extent to which delirium could be a modifiable risk factor for dementia.

Our data should be interpreted in the context of some limitations. Although recruited participants closely match the sampling frame by age and socioeconomic position (but not ethnicity), the overall response rate was low and other reasons for non-participation are unknown. Despite real-time access to health records, we relied on self-reporting for many of the variables, and telephone-based cognitive assessment cannot test some domains such as visuospatial function. Although we had comprehensive methods to identify those participants admitted to hospital, there is inevitably a degree of selection bias that would have missed participants who developed delirium but stayed in the community. Furthermore, we could not accurately establish delirium duration if patients were discharged because their condition was persistent. Despite the advantage of frequent clinical assessments, we made assumptions about missing data on delirium status over weekends and public holidays. There was appreciable loss to follow-up, although because this was more likely in those with poorer baseline cognition, we might have underestimated the effect of delirium on cognitive outcomes. We did not account for medication-related effects, nor did we explore possible differences attributable to underlying causes; these are areas of ongoing analysis. In common with other observational studies, model estimates are subject to residual confounding, particularly if there are relevant quantities not captured by our frailty index. Nonetheless, the prospective assessment of brain symptoms before and during acute illness allows for the most rigorous mapping of baseline cognition, admission to hospital, and delirium in a community sample to date.

Overall, these data hold several implications for clinical care. On hospital admission, establishing pre-admission baseline cognitive function is informative in predicting if the risk is mainly for long-term delirium (low baseline cognition) or for significant cognitive decline or death (high baseline cognition). If delirium is slow to resolve in a patient with high baseline cognition, the data presented here suggest a need to enhance care, including managing underlying precipitating and perpetuating factors. However, delivering this management for better patient care requires urgent consideration of delirium implementation practice, where we are far from systematically embedding delirium detection and recovery measures.^30^ Moreover, because baseline cognition appears to be such a powerful predictor of the course of acute illness, efforts to digitally integrate cognitive information from other settings, such as primary care or memory clinics, should be prioritised. The present findings also provide novel information in a cohort of emergency admissions regarding the risks of future cognitive decline, especially in people without substantial pre-admission cognitive impairment. Ultimately, ongoing DELPHIC investigations will further elucidate if any phenomenological or causal components are linked with specific clinically relevant outcomes (eg, cognitive function, frailty, and mortality), as well as the broader relationships with subcomponents of our baseline cognitive measures (eg, episodic memory and verbal fluency). As this understanding translates into more precise estimates of delirium risk, we will be able to offer much more tailored advice for patients across the spectrum of cognitive function.

In conclusion, higher baseline cognitive function distinguishes those who are likely to have shorter and less severe delirium. Yet individuals with higher baseline cognition who develop delirium are disproportionately susceptible to subsequent cognitive impairment after delirium, experiencing more than 1 SD decline in general cognition over 2 years.

## Figures and Tables

**Table 1:** Characteristics of the cohort in relation to admission to hospital and delirium status

	Whole cohort, n=1510	People who experienced delirium		Lost to follow-up		Died		Followed up	
		n=115	p value	n=199	p value	n=93	p value	n=1218	p value
Age (years)	78 (6·2)	82 (6·6)	<0·0001	80 (6·8)	<0·0001	83 (5·8)	<0·0001	77 (5·6)	0·57
Sex	..	..	..	..	..	..	..	..	<0·0001
Women	865 (57%)	63 (55%)	0·58	119 (60%)	0·36	43 (46%)	0·041	706 (58%)	..
Men	645 (43%)	52 (45%)	..	80 (40%)	..	50 (54%)	..	512 (42%)	..
Education	..	..	<0·0001	..	<0·0001	..	<0·0001	..	<0·0001
Degree-level	982 (65%)	46 (40%)	..	107 (54%)	..	37 (40%)	..	828 (68%)	..
Up to secondary	317 (21%)	35 (30%)	..	50 (25%)	..	25 (27%)	..	244 (20%)	..
Up to primary	211 (14%)	34 (30%)	..	42 (21%)	..	31 (33%)	..	146 (12%)	..
Ethnicity	..	..	..	..	..	..	..	..	0·26
White	1419 (94%)	102 (89%)	0·044	181 (91%)	0·12	88 (95%)	0·89	1157 (95%)	..
Other	91 (6%)	13 (11%)	..	18 (9%)	..	5 (5%)	..	61 (5%)	..
Frailty index	0·15 (0·13)	0·30 (0·17)	<0·0001	0·2 (0·17)	<0·0001	0·30 (0·1)	<0·0001	0·13 (0·1)	<0·0001
Modified Telephone Interview for Cognitive Status (total)	38·8 (5·9)	33·8 (8·7)	<0·0001	36 (7·5)	<0·0001	34 (4·9)	<0·0001	40 (4·9)	<0·0001
Fluency (words)	15·6 (6·2)	11·6 (6·8)	<0·0001	14 (6·1)	<0·0001	11 (6·8)	<0·0001	16 (6·0)	<0·0001
Fluency (animals)	19·0 (7·0)	13·3 (7·4)	<0·0001	17 (7·5)	<0·0001	13 (6·5)	<0·0001	20 (6·5)	<0·0001
Self-rated health	..	..	..	..	..	..	..	..	<0·0001
Poor or very poor	272 (18%)	56 (49%)	<0·0001	48 (24%)	<0·0001	43 (46%)	<0·0001	171 (14%)	..
Good, very good, or excellent	1238 (82%)	59 (51%)	..	151 (76%)	..	50 (54%)	..	1047 (86%)	..
Past medical history	..	..	..	..	..	..	..	..	<0·0001
Myocardial infarction	317 (21%)	43 (37%)	<0·0001	44 (22%)	0·59	33 (36%)	<0·0001	238 (20%)	0·36
Diabetes	181 (12%)	22 (19%)	0·019	30 (15%)	0·17	23 (25%)	<0·0001	130 (11%)	0·30
Hypertension	755 (50%)	70 (61%)	0·026	96 (48%)	0·61	62 (67%)	<0·0001	378 (31%)	0·11
Stroke	136 (9%)	18 (16%)	0·030	22 (11%)	0·34	19 (20%)	<0·0001	97 (8%)	<0·0001
Cancer	362 (24%)	29 (25%)	0·64	38 (19%)	0·13	27 (29%)	0·19	292 (24%)	0·49
Chronic obstructive pulmonary disease	211 (14%)	32 (28%)	<0·0001	34 (17%)	0·24	26 (28%)	0·001	146 (12%)	<0·0001
Any impaired personal activities of daily living*	136 (9%)	36 (31%)	<0·0001	36 (18%)	<0·0001	33 (36%)	<0·0001	73 (6%)	<0·0001
Any impaired instrumental activities of daily living†	1102 (73%)	104 (90%)	<0·0001	153 (77%)	<0·0001	80 (86%)	<0·0001	889 (73%)	<0·0001

Data presented as mean (SD) or n (%). p values refer to the following comparisons: delirium compared with the whole cohort; attrition compared with those followed up; died compared with those followed up; and followed up compared with the whole cohort. *Personal activities of daily living include: grooming, toileting, dressing, bathing, transfer, and stairs. †Instrumental activities of daily living include: shopping, washing up, making hot drinks, feeding, and walking outside.

**Table 2:** Associations between delirium burden and follow-up cognition

	Follow-up cognition (n=1218)				Mortality model (n=1510)			
	β	95% CI		p value	Hazard ratio	95% CI		p value
Delirium burden	..	..	..	<0·0001	..	..	..	<0·0001
None	Ref	..	..	..	Ref	..	..	..
Low	0·03	–0·20	0·26	..	7·14	3·50	14·6	..
High	–0·60	–0·93	–0·26	..	13·74	6·75	28·0	..
Baseline cognition	0·58	0·51	0·65	<0·0001	0·64	0·49	0·83	<0·0001
Interaction between delirium and baseline cognition	..	..	..	<0·0001*	..	..	..	0·016*
None	Ref	..	..	..	Ref	..	..	..
Low	0·08	–0·11	0·26	..	1·53	0·93	2·51	..
High	–0·35	–0·56	–0·14	..	1·59	1·14	2·20	..
Education	..	..	..	<0·0001*	..	..	..	0·35*
Up to primary	Ref	..	..	..	Ref	..	..	..
Up to secondary	0·12	–0·05	0·29	..	1·00	0·57	1·74	..
Degree-level	0·30	0·14	0·45	..	0·71	0·42	1·20	..
Age	–0·02	–0·03	–0·01	<0·0001	1·27	1·02	1·57	0·030
Sex	0·05	–0·04	0·14	0·26	0·66	0·44	1·01	0·057
Frailty index	–0·06	–0·13	0·01	0·079	1·18	0·98	1·42	0·086
National Early Warning Score	0·01	–0·06	0·08	0·83	1·13	0·99	1·29	0·063

Baseline and follow-up cognition were derived using the modified Telephone Interview for Cognitive Status plus two verbal fluency measures (per SD): frailty index, minus the cognitive items to avoid collinearity; and the national early warning score, calculated as mean over study admission days. Low and high delirium burden was defined by taking the median of the cumulative Memorial Delirium Assessment Scale scores. Multivariable analyses show coefficients mutually adjusted for all other factors. Baseline cognition, frailty index, and mean national early warning score were standardised per SD, and age is represented per year. *p value for trend.

## Data Availability

Complete deidentified participant data, along with study protocols, consent forms, and case report forms are available through the Dementias Platform UK Data Portal: https://portal.dementiasplatform.uk/.

## Research in context

### Evidence before this study

We searched MEDLINE and Embase, updating our previous systematic reviews. Our search terms were “Delirium” (title) AND (“epidemiology” OR “prevalence” OR “incidence” OR “occurrence”) (title/abstract) for records up to Sept 8, 2021 (updating a search from May 31, 2019). All languages were included in the search and we included studies with a population-based sampling frame. Apart from our related Delirium and Cognitive Impact in Dementia study, we found no population studies on long-term cognitive impairment after delirium that could account for both baseline cognition and delirium severity and duration.

### Added value of this study

To the best of our knowledge, this is the first study to prospectively follow a community sample before, during, and after an acute illness. This method overcomes the limitations of previous study designs by allowing for the quantification of cognitive decline observed after admission to hospital and of delirium in relation to baseline cognition.

### Implications of all the available evidence

In patients without significant cognitive impairment, delirium is an important indicator for marked decline in future cognition and the insult required to develop delirium in such patients might be particularly dangerous. Establishing baseline cognitive function is necessary to differentiate groups at risk of severe or long-term delirium (lower baseline cognition) or long-term cognitive impairment (higher baseline cognition). If delirium appears to be slow to resolve in a patient with higher baseline cognition, the natural history described in the current study suggests the potential need to enhance care during and after delirium. People without significant baseline cognitive impairment who develop delirium should be provided with information about the risk of significant cognitive decline in the 2 years after the episode of delirium.
