# Supplementary material for: The effect of baseline cognition and delirium on long-term cognitive impairment and mortality: a prospective population-based study
Source: Lancet Healthy Longev. 2022 Apr;3(4):e232–41. doi: 10.1016/S2666-7568(22)00013-7 (PMC7612581; doi:10.1016/S2666-7568(22)00013-7)

THE LANCET
Healthy Longevity

Supplementary appendix

This appendix formed part of the original submission and has been peer reviewed. We post it as supplied by the authors.

Supplement to: Tsui A, Searle SD, Bowden H, et al. The effect of baseline cognition and delirium on long-term cognitive impairment and mortality: a prospective population-based study. *Lancet Healthy Longev* 2022; published online March 15. https://doi.org/10.1016/S2666-7568(22)00013-7.

Supplementary Table 1. Demographic characteristics of the DELPHIC sample in relation to the London Borough of Camden

	**Camden**	**DELPHIC**
Age (median, IQR)	77 (73-83)	77 (73-82)
Female	58%	57%
Index of multiple deprivation (median, IQR)	18·9 (11·3-28·4)	14·6 (9·1-22)
Ethnicity (% white)	84%	94%

Supplementary table 2. The association between baseline cognition and delirium prevalence, severity and duration.

Inpatient days = 1999		**Point prevalence**				**Severity**				**Duration**		
Number of groups = 209	**OR**	**95%CI**		**P**	**β**	**95%CI**		**P**	**IRR**	**95%CI**	**P**	**IRR**
Baseline cognition	0.63	0.45	0.89	0.009	-1.60	-2.55	-0.66	0.001	0.88	0.77	1.00	0.054
Education				0.59				0.53				0.83
Up to primary	Ref				Ref				Ref			
Up to secondary	1.49	0.67	3.28		1.19	-0.95	3.33		1.14	0.74	1.78	
Degree level	1.09	0.52	2.26		0.82	-1.16	2.80		1.09	0.72	1.63	
Age (per year)	0.98	0.70	1.38	0.90	0.03	-0.10	0.17	0.66	1.00	0.98	1.03	0.79
Sex	0.90	0.50	1.61	0.72	-1.35	-2.91	0.22	0.092	0.96	0.69	1.33	0.81
Frailty Index (per SD)	1.27	0.89	1.80	0.18	0.41	-0.57	1.38	0.41	1.06	0.92	1.23	0.40
NEWS (per point)	0.89	0.66	1.22	0.48	0.08	-0.10	0.25	0.39	0.95	0.86	1.04	0.27

Mixed-effects logistic regression accounts for repeated measures per individual. Baseline cognition derived from modified Telephone Interview of Cognitive Status plus two verbal fluency measures (per SD); FI frailty index, minus cognitive items to avoid collinearity; NEWS national early warning score. Univariable analyses are individual models per row; multivariable analyses show coefficients mutually adjusted for all other factors.

Supplementary Figure 1. Distribution of age and index of multiple deprivation comparing the London Borough of Camden with the DELPHIC sample

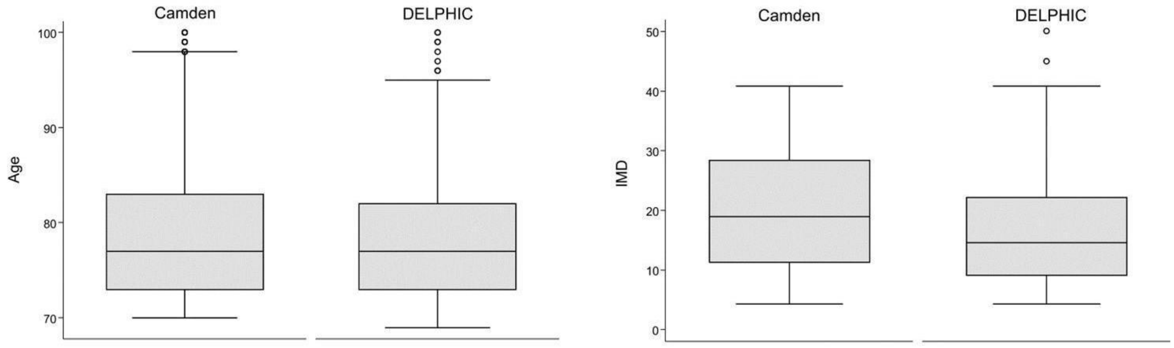

Supplement: Supplementary appendix [file mmc1.pdf]
